# Multi-Parameter Detection of Urine Based on Electropolymerized PANI: PSS/AuNPs/SPCE

**DOI:** 10.3390/bios13020272

**Published:** 2023-02-14

**Authors:** Dong Wang, Xiyu Mao, Yitao Liang, Yu Cai, Tingting Tu, Shanshan Zhang, Tianyu Li, Lu Fang, Yue Zhou, Zhaoyang Wang, Yu Jiang, Xuesong Ye, Bo Liang

**Affiliations:** 1 Biosensor National Special Laboratory, Key Laboratory of Biomedical Engineering of Ministry of Education, College of Biomedical Engineering and Instrument Science, Zhejiang University, Hangzhou 310027, China; 2 Department of General Surgery, Sir Run Run Shaw Hospital, School of Medicine, Zhejiang University, Hangzhou 310027, China; 3 College of Automation, Hangzhou Dianzi University, Hangzhou 310018, China; 4 Binjiang Institute of Zhejiang University, Hangzhou 310053, China

**Keywords:** electrochemical biosensor, PANI: PSS, ammonium ion, urea, creatinine, urinalysis

## Abstract

Urine analysis is widely used in clinical practice to indicate human heathy status and is important for diagnosing chronic kidney disease (CKD). Ammonium ions (NH_4_^+^), urea, and creatinine metabolites are main clinical indicators in urine analysis of CKD patients. In this paper, NH_4_^+^ selective electrodes were prepared using electropolymerized polyaniline-polystyrene sulfonate (PANI: PSS), and urea- and creatinine-sensing electrodes were prepared by modifying urease and creatinine deiminase, respectively. First, PANI: PSS was modified on the surface of an AuNPs-modified screen-printed electrode, as a NH_4_^+^-sensitive film. The experimental results showed that the detection range of the NH_4_^+^ selective electrode was 0.5~40 mM, and the sensitivity reached 192.6 mA M^−1^ cm^−2^ with good selectivity, consistency, and stability. Based on the NH_4_^+^-sensitive film, urease and creatinine deaminase were modified by enzyme immobilization technology to achieve urea and creatinine detection, respectively. Finally, we further integrated NH_4_^+^, urea, and creatinine electrodes into a paper-based device and tested real human urine samples. In summary, this multi-parameter urine testing device offers the potential for point-of-care testing of urine and benefits the efficient chronic kidney disease management.

## 1. Introduction

Among the existing chronic diseases, chronic kidney disease (CKD) has a high incidence and a high mortality rate because CKD becomes progressively more severe and has an increased risk of complications if patients do not intervene in the long term, so it is highly necessary to diagnose and manage CKD as early as possible [1]. If renal function is further impaired, patients may require dialysis or even kidney transplantation. To improve their quality of life, dialysis frequency is critical for patients requiring dialysis and requires frequent testing of serum metabolites, such as creatinine, urea, and potassium levels. However, frequent venipuncture will increase the risk of infection in patients [2,3], it has been reported that the detection of relevant metabolites in urine can be used as an alternative to blood testing.

Blood creatinine levels are a major indicator for assessing renal function, thyroid dysfunction, and muscle damage [4,5]. Renal function is usually clinically assessed in combination with blood urea nitrogen testing [6]. However, the reflection of blood creatinine and blood urea nitrogen on renal function impairment is lagging and can only be reflected by severe impairment, making it difficult to achieve early identification of renal disease. To reduce the impact of this lag, the degree of renal impairment can be reflected by testing the blood creatinine-urinary creatinine ratio and calculating creatinine clearance [7,8], and blood creatinine and creatinine clearance are important indicators for evaluating the glomerular filtration rate [9]. Acid removal by the kidneys is a critical procedure in maintaining human acid-base balance [10], and it metabolizes the daily acid load by excreting ammonium ions (NH_4_^+^) and titratable acids [11]. Metabolic acidosis may result when there is an increase in acid production by the kidneys or a decrease in the amount of acid excreted [12]. In patients with CKD, the progressive decrease in renal function results in decreased total urinary NH_4_^+^ (uNH_4_^+^). The excretion of urinary NH_4_^+^ decreases in parallel with the glomerular filtration rate, while the excretion of titratable acid is maintained until the late stages of CKD [13]. The incidence of metabolic acidosis (usually defined as total serum carbon dioxide (tCO_2_) of 22 mEq/L) is about 15% in patients with CKD who do not require dialysis [14,15]. Since uNH_4_^+^ excretion is essential to maintain a normal tCO_2_, uNH_4_^+^ may be an earlier risk indicator than tCO_2_ and even an independent predictor of deteriorating renal function [16]. In conclusion, for CKD patients, detecting creatinine, urea, and NH_4_^+^ in urine is crucial.

The main methods of detecting NH_4_^+^ are the ion electrode method, the nano reagent colorimetric method [17], the titration method [18], and the ion chromatography method [19]. Nano reagent colorimetric is a usual clinical method. However, its measurement results are easily affected by the colored ions and turbidity in the system to be measured. Traditional urea testing methods, such as chromatography and colorimetric methods, face challenges such as high equipment costs, specialized personnel, and long analysis times [20]. The colorimetric method based on chemical reactions is the main method to detect creatinine, but it lacks specificity and is easily interfered by various metabolites in body fluids [21]. Electrochemical detection methods based on NH_4_^+^-selective electrodes have been widely studied because of their rapid, convenient, non-toxic, and non-polluting characteristics, and the detection of both creatinine and urea can be achieved by immobilizing the corresponding enzymes on NH_4_^+^-selective electrodes. Electrochemical sensors for detecting NH_4_^+^ are usually used to determine the amount of NH_4_^+^ by measuring the change in electrode potential [22,23]. The disadvantage is that the ionic strength of the measured solution affects the membrane potential [24], which ultimately reduces the measurement accuracy. Instead, the method of measuring NH_4_^+^ by measuring the current response offers a faster response, higher linearity and sensitivity, and the advantage of resisting the effect of ionic strength compared to potentiometric sensors [25].

Conducting polymers (CPs) have been widely used in several fields (energy storage devices, electrochemical sensors, drug delivery systems) due to their simple preparation and good stability [26,27]. PANI is one of the most reported CPs to date and has a wide range of applications in developing electrochemical biosensors due to its multiple oxidation states, more dispersive redox potential, excellent electrical conductivity, and good biocompatibility [28,29]. For example, a non-enzymatic sensor of hydrogen peroxide with Ag nanoparticles-PANI nanotubes (AgNPs-PANINTs) composites [30], and biosynthetic nanocomposites (B-CuFeO2/PANI NCs) for antibacterial and quantifies hydrazine in agricultural applications [31]. PANI has unique selectivity for ammonia (NH_3_), and is therefore widely used for NH_3_ detection [32,33]. In acidic environments, PANI is able to form a stable structure with positive nitrogen ions through the processes of protonation and depolarization. When there is NH_3_ in the environment, due to the electron-giving effect of NH_3_, it can take away the hydrogen ions on the aniline to form NH_4_^+^, forming hydrogen bonds between NH_4_^+^ and the nitrogen in the aniline. The negatively charged anion group (A^−^) in the PANI skeleton interacts with the positively charged NH_4_^+^ with a charge to form a stable doped structure, and the number of carriers decreases due to the transfer of polaritons in the PANI skeleton to NH_3_; the resistance of PANI increases. On the contrary, when air is passed, the reaction equilibrium shifts to the left and PANI changes back to the doped state, which shows a decrease in resistance [34]. PANI can be modified with Perfluorosulfonic acid (Nafion) to provide sulfonic acid groups during PANI doping/dedoping as a way to detect NH_4_^+^ [35,36]. Zhybak prepared a PANI-Nafion-Cu based screen printed electrode for urea and creatinine detection [37], but it was limited by its dependence on the electrocatalytic reaction between NH_4_^+^ and metal/nanocatalysts. Uzunçar and his colleagues prepared the nanopolymer PANI: PSS by chemical polymerization and then modified the glassy carbon electrode (GCE) with Nano-PANI: PSS to achieve an amperometric NH_4_^+^ detection sensor [25]. Furthermore, gold nanoparticles (AuNPs) are widely used in electrochemical biosensors due to their low cytotoxicity and strong affinity for various enzymes and thiol- or amine-containing molecules, such as proteins [38]. AuNPs increase the reaction surface area of the sensor, improve the electron transfer efficiency, amplify the signal, and obtain high sensitivity [39].

**Figure 1:**
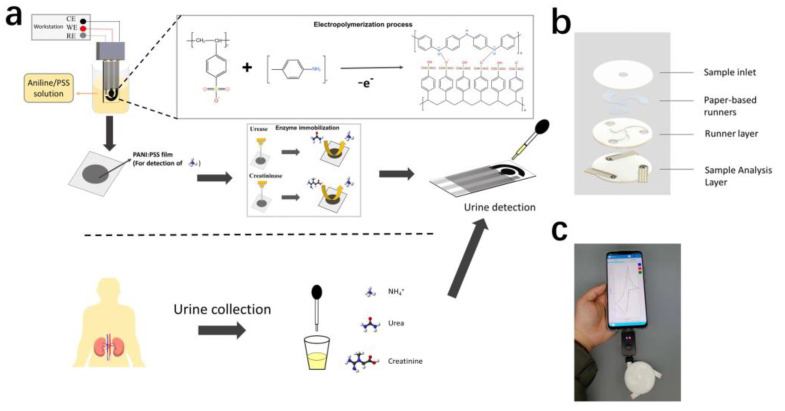
(**a**) The screen-printed electrode (SPCE) was modified with PANI: PSS to detect NH_4_^+^, and immobilized urease and creatinine deiminase to detect urea and creatinine in urine; (**b**) Urine multi-parameter detection sensor structure schematic; (**c**) Multi-parameter urine testing system, including smartphone, handheld electrochemical detection device, and this urine multi-parameter testing device.

In this paper, we proposed a controlled electropolymerization approach to modify PANI: PSS film as a NH_4_^+^-sensitive material on screen-printed carbon electrodes (Figure 1a). Before modifying the electropolymerized PANI: PSS, we modified a layer of AuNPs on the screen-printed electrode to enhance the sensor performance. The electro-polymerized PANI: PSS film exhibited a high selectivity for NH_4_^+^ when a redox reaction occurred and had a wide linear detection range, which could be used for detecting uNH_4_^+^. On this basis, the urea and creatinine electrodes with wide detection range were further prepared by modifying urease and creatinine deiminase upon the electropolymerized PANI: PSS film based on ammonium detection using the enzyme immobilization technique. For the interference of endogenous NH_4_^+^, an anion exchange membrane was further modified on the enzyme electrode, which effectively reduced the influence of endogenous NH_4_^+^ on the enzyme electrode. Finally, we designed a paper-based integrated multiparameter assay (Figure 1b), which could be used for cell phone POCT assay in the future (Figure 1c).

## 2. Materials and Methods

### 2.1. Reagents and Instruments

#### 2.1.1. Reagents

Anion exchange membrane (FAA-3-SOLUT-10, 5% NMP solution) was purchased from FuMA-Tech. Ammonium chloride, Chloroauric acid, Bovine serum protein (BSA), and ammonium persulfate were purchased from Aladdin Reagent Co (Shanghai, China). Phosphate buffer solution (0.01 M PBS, pH = 7.2~7.4), urease (251 U/mg), and creatinine deiminase (10 U/mg) were purchased from Shanghai Yuanye Biological (Shanghai, China); other reagents were purchased from Sinopharm Reagent Co (Shanghai, China). The experimental water was deionized water (18 MΩ/cm).

#### 2.1.2. Instrument

Electrochemical workstation (μAutolab III, Metrohm, Herisau, Switzerland) and its supporting software NOVA; field emission scanning electron microscope (SU-8010, Hitachi, Tokyo, Japan); FTIR spectroscopy (characterization using (Nicolet iS 50, Thermo Fisher, Shanghai, China); 120 kV transmission electron microscope (HT-7700, Hitachi).

### 2.2. Preparation of PANI: PSS-Based NH_4_^+^-Selective Electrodes

The screen-printed electrodes were ultrasonically cleaned in deionized water for 5 min, dried, and set aside. The electrode was immersed in the prepared solution (10 mM HAuCl_4_,0.5 M H_2_SO_4_), and the nanogold was reduced on the electrode by constant pressure plating (0 V, 60 s). The electrode was then placed in the plating solution (1 M HCL, 0.3 M aniline, 12 mg/mL PSS), and the PANI: PSS film was assembled onto the working electrode by cyclic voltammetry (range: −0.2 V~1 V, rate: 0.1 V/s, 10 cycles).

### 2.3. Preparation of Urea Electrode and Creatinine Based on NH_4_^+^ Detection

Based on the NH_4_^+^ detection electrode in Section 2.2, 2 μL of urease solution (20 mg/mL urease, 5 mg/mL BSA, 0.01 M PBS), or 2 μL of sarcosine anhydrase (CD) solution (30 mg/mL CD, 10 mg/mL BSA, 0.01 M PBS) was dropped onto the working electrode surface in the first step, and then dried in a refrigerator at 4 °C. In the second step, 50 μL of 25% glutaraldehyde was dropped around the working electrode to cross-link the enzyme in the gas phase (60 min in a 37 °C thermostat). Finally, 2 μL of anion exchange membrane solution (2.5% aqueous solution) was added dropwise on the surface of the enzyme electrode, and dried in the refrigerator at 4 °C for backup.

### 2.4. Test Method for Electrochemical Characteristics of Electrodes

The standard curves of the three electrodes were tested by cyclic voltammetry (in 0.01 M PBS, scan range −0.8 V~0.8 V, scan speed 100 mV/s, 8 turns). Three parallel experiments were performed using the same electrode, and the eighth turn of oxidation peak current was taken to obtain the corresponding standard curves. The test method for electrode selectivity is provided in the illustration of the figure. Test methods for consistency and stability of electrodes are provided in the Supplementary Materials.

## 3. Results and Discussion

### 3.1. Characterization of PANI: PSS Films

#### 3.1.1. Scanning Electron Micrograph of PANI: PSS Film

**Figure 2:**
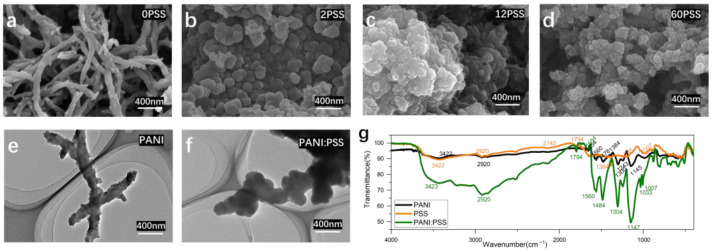
(**a**–**d**) the SEM images of PANI: PSS electroplating doped with different concentrations of PSS (Unit: mg/mL); (**e**,**f**) the TEM images of PANI without PSS doping and PANI: PSS (PSS:12 mg/mL); (**g**) infrared absorption spectrum of PANI, PSS, and PANI: PSS.

As in Figure 2a, the surface morphology of the electropolymerized PANI electrode is a striped mesh of polyaniline with a diameter of about 100 nm, similar to previous studies [40]. However, the surface structure of the electropolymerized PANI: PSS electrodes is a stacked particle structure, which is different from the chemically synthesized PANI: PSS [25]. This is because PSS can be regarded as a kind of macromolecular acid, which modifies the electrode surface together with PANI in the process of PANI polymerization and has a great influence on the morphological changes during the growth of PANI. The polymerization process of PANI starts as tiny spherical particles; after doping by hydrogen ions in hydrochloric acid solution, the surface is positively charged and has a heterogeneous charge attraction with the negatively charged PSS long chain with abundant sulfonic acid groups on the surface, thus affecting the structural growth of PANI. In Figure 2b, the electrode surface containing 2 mg/mL PSS still shows the presence of partially streaked structures (particle size is around 150 nm); looking at Figure 2c,d, when the concentration of PSS increases, only particle accumulation structures remain on the electrode surface, and the average diameter of spherical particles decreases with further increases in concentration.

#### 3.1.2. Transmission Electron Micrograph (TEM) of PANI: PSS Film

Figure 2e shows the transmission electron microscopy image of polyaniline without PSS doping as long strips with particles around 100 nm in diameter, similar to the SEM image results of 0 mg/mL PSS shown in Figure 2a. Figure 2f shows the TEM image of PSS concentration of 12 mg/mL PSS in the plating solution, and the morphological structure is similar to the SEM image in Figure 2c, both of which are agglomerates of stacked particles. Compared with the image of pure PANI, the PANI: PSS image shows a bulkier particle agglomeration, which proves that PANI and PSS polymerize into spherical particles at the same time during the plating process, and the two are more tightly bound.

#### 3.1.3. Fourier Infrared Spectroscopy (FTIR) of PANI: PSS Films

FTIR characterization was performed for PANI, PANI: PSS, and PSS, respectively. As shown in Figure 2g, the FTIR spectra of PANI, PSS, PANI: PSS all show broad peaks of N-H stretching vibrations from PANI at 3422 cm^−1^ and C-H stretching vibrations at 2920 cm^−1^. The peak at 1560 cm^−1^ in the PANI, PANI: PSS spectra indicates the stretching vibrations of the quinone and benzene rings at 1478 cm^−1^ and 1484 cm^−1^, respectively. The intensity ratio of the stretching vibration peaks of the quinone ring and benzene ring can be used to reflect the degree of oxidation of PANI. The sharp peak near 1654 cm^−1^ is due to the stretching vibration of the C=N double bond on the benzene ring; 1243 cm^−1^ is the stretching vibration of the C-H single bond on the benzene ring. At 1145 cm^−1^ and 1147 cm^−1^ are the bending vibrations of the C-H single bond on the quinone ring. The characteristic absorption peaks of the sulfonic acid group are at 1205 cm^−1^ and 1007 cm^−1^ of the absorption spectra of PSS, and the PANI: PSS spectra show the same characteristic absorption peaks of the sulfonic acid group at 1033 cm^−1^ and 1007 cm^−1^. The above results provide evidence for the structure of polyaniline, which is consistent with that proposed in the previous work [41].

### 3.2. Performance Testing and Mechanism Investigation of NH_4_^+^-Selective Electrode Based on PANI: PSS

#### 3.2.1. Performance Test

**Figure 3:**
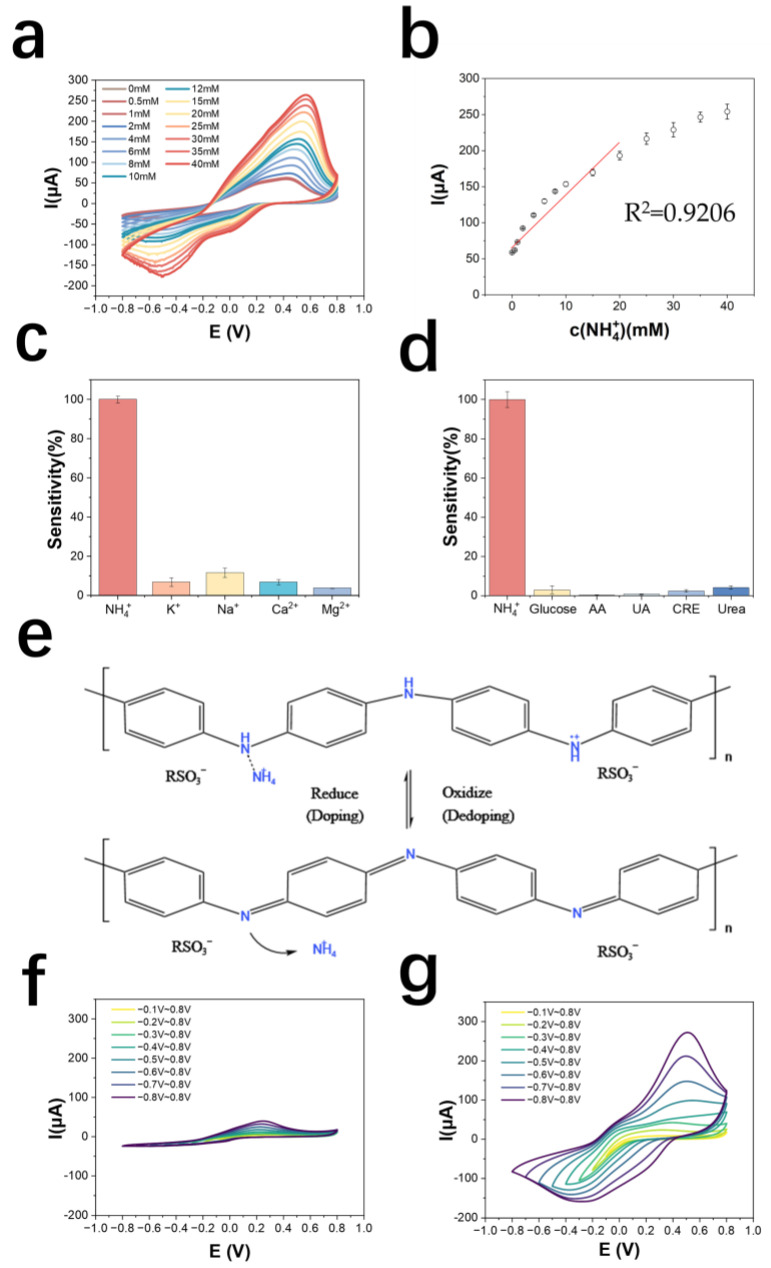
(**a**) Cyclic voltammetric curves of PANI: PSS in solutions with different concentrations of ammonium chloride; (**b**) NH_4_^+^ detection calibration curve; (**c**) selectivity of PANI: PSS electrode for cations (the added cations were all at a concentration of 5 mM); (**d**) selectivity of PANI: PSS electrode for metabolites (n = 3) (The electrode was immersed in 0.01 M PBS solution and cyclic voltammetric scanning was performed. 5 mM NH_4_Cl and common metabolite solutions including 50 μM glucose (Glucose), 100 μM ascorbic acid (AA), 150 μM uric acid (UA), 1 mM creatinine (CRE), and 10 mM urea (Urea) were sequentially added; cyclic voltammetric scans were performed again after each addition of interfering metabolite solutions. The absolute value of the change in the oxidation peak current of the cyclic voltammetric curve is the interference brought by the interfering metabolite); (**e**) Dynamic doping/dedoping process of PANI: PSS by NH_4_^+^; (**f**) 0.01 M PBS in dynamic reduction potential cyclic voltammetric curve; (**g**) dynamic reduction potential cyclic voltammetric curve in 5 mM ammonium chloride solution.

The detection and standard curves of NH_4_^+^ are shown in Figure 3a,b, which can be derived as follows: the detection range is 0.5~40 mM, the linear range is 0.5~20 mM, the detection limit is 290.1 μM (LOD = 3σ/S; σ: the standard deviation of measuring blank value; S: Slope of the standard curve). The calibration curve equation is y = 9.45x + 63.86 (y is μA, x is mM), the correlation coefficient is 0.921, and the sensitivity of the electrode is 192.6 mA M^−1^ cm^−2^. The results of normalizing the current values of the electrode to the cation response are shown in Figure 3c, indicating that the AuNPs/PANI: PSS electrode has good selectivity for NH_4_^+^. As shown in Figure 3d, the electrode has good metabolite immunity to glucose, ascorbic acid, uric acid, creatinine, and urea, all with responses lower than 5% of the response to NH_4_^+^. Compared to other NH_4_^+^ sensors made of PANI-doped materials, this sensor has a larger linear range while maintaining a higher sensitivity (Table S5). Table 1 shows a comparison of this work with other work on NH_4_^+^ detection electrodes. Compared to other work, the electropolymerized PANI: PSS electrode has a wide detection range while maintaining high sensitivity.

#### 3.2.2. Exploration of the Mechanism

To investigate the mechanism of PANI: PSS selectivity for NH_4_^+^, a control test was conducted in this paper. A polyaniline electrode polymerized with the same cyclic voltammetry scan method using a PSS-free plating solution as a control was tested in solution, and the results are shown in Figure S7a. In the 0.01 M PBS solution (pH = 7.2~7.4), there was only about 5 μA of current and no redox peak between −0.8 V~0.8 V. The current of cyclic voltammetry did not significantly change after the addition of ammonium chloride, proving that the non-PSS-doped PANI did not respond to NH_4_^+^. On the contrary, the experimental results of the electrode for electro-polymeried PANI: PSS (Figure 3a), when the concentration of NH_4_^+^ in the solution increases, there is a clear increase of redox current; meanwhile, even in 0.01 M PBS without NH_4_^+^, a clear redox peak can be observed in the curve of PANI: PSS, as shown in Figure S7b, which proves that the PANI: PSS electrode has redox activity in the PBS solution and a redox reaction occurs.

PSS is a polymer with a large number of sulfonic acid groups that can gather the hydrogen ions in solution, resulting in a localized pH around the PSS that is less than the pH of the solution as a whole [46]. When PSS is doped with PANI, the enriched hydrogen ions around PSS are utilized by PANI, making PANI: PSS a hydrogen-ion-doped state with electrical conductivity, as shown in Figure S8. When the content of PSS around PANI increases, due to the increase in enriched hydrogen ions, the vacancy of doping sites on PANI decreases, and the existence of spatial site resistance makes the doping of NH_4_^+^ more difficult, so only a moderate ratio of PANI to PSS shows the maximum detection sensitivity, which is consistent with the experimental results in Figure S4 and Table S3.

To further demonstrate the effect of environmental hydrogen ions on PANI activity, NH_4_^+^ detection scans were performed using a PANI electrode without PSS in a McIlvaine buffer solution at pH = 3, pH = 5. The detection method was cyclic voltammetry with a scan range of −0.8 V~0.8 V and a scan number of five turns. The results are shown in Figure S9a. In pH = 3, pH = 5 buffer solution, a clear reduction peak with increasing NH_4_^+^ concentration was observed, but no oxidation peak, which indicates that PANI also respondeed to NH_4_^+^ at a lower pH environment, and the current magnitude at pH = 3 was lower than that at pH = 5, indicating that the selectivity of PANI for NH_4_^+^ was related to the pH value in the environment related; the lower the pH, the higher the hydrogen ion doping of PANI, the higher the electrochemical activity, and the lower the peak potential. The scan range was further extended to probe the position of the oxidation peak (Figure S9b); e.g., after increasing the upper scan limit of the oxidation potential to 1.0 V, 1.2 V, and 1.5 V, the position of the oxidation peak could be observed around 1.0 V. However, too high a positive potential would break the PANI chain and could not be used for the detection of NH_4_^+^.

As shown in Figure S10a, 5 mM ammonium chloride was added to the solution every 50 s in a solution of 0.01 M PBS, and measured and recorded the change in open circuit potential between the working electrode and reference electrode, and it was obvious that the open circuit potential did not significantly change and was stable at about 0.124 V over a period of 300 s. Figure S10b shows the time-current curve obtained by adding 5 mM ammonium chloride to the solution at an interval of about 50 s after applying a bias voltage of −0.2 V for 300 s, and recording the time—current curve. It can be seen that PANI: PSS has an immediate response to the change in NH_4_^+^ concentration in the solution with a current increase of about 3 μA, and the change in current is much smaller than the change in peak current (about 50 μA) in cyclic voltammetry. Moreover, the current response gradually decreases to the original level within 30 s. Combining the experimental results in Figure 3a, the detection of NH_4_^+^ by PANI: PSS depends on the change of applied potential, so the open-circuit potential detection without applied potential and amperometric method with applied constant bias potential cannot be used for the detection of NH_4_^+^.

From the above experimental results, it can be inferred that the electro-polymeried PANI: PSS electrode exhibits selectivity for NH_4_^+^ under the condition of local enrichment of hydrogen ions subjected to changes in the applied potential causing the redox reaction of PANI. The enrichment of hydrogen ions by PSS makes some of the doping sites of PANI doped by hydrogen ions and obtain electrochemical activity, at this time the doping sites of polyaniline are still vacant, and NH_4_^+^ in solution can dope PANI, which makes the number of carriers of PANI increase, and the conductivity is further improved, which shows the increase in redox current, and the dynamic doping/dedoping of NH_4_^+^ by PANI: PSS. The mechanism is shown in Figure 3e. The process of doping/dedoping of PANI: PSS by NH_4_^+^ can be shown by Equation (1), and it can be considered that PANI: PSS has dynamic selectivity for NH_4_^+^.

PANI_OX_: PSS + NH_4_^+^ + e^−^ ⇌ [PANI_RE_: NH_4_^+^]: PSS (1)

To verify the dynamic doping/dedoping effect of NH_4_^+^ on PANI: PSS, CV scans were performed with electrodes in 0.01 M PBS solution and 0.01 M PBS solution containing 5 mM ammonium chloride at different voltage ranges, respectively. The range of oxidation potential was first kept constant and the lower limit of the scan of reduction potential was continuously increased from −0.1 V to −0.8 V. The scans in 0.01 M PBS solution are shown in Figure 3f, and those in 0.01 M PBS solution containing 5 mM ammonium chloride are shown in Figure 3g. As the reduction potential in the voltage range of the cyclic voltammetric scan gradually increases, the current of the oxidation peak also gradually increases. The reduction state of polyaniline increases and the electrode has the ability to dope more NH_4_^+^, which shows an increase in reduction current, demonstrating the dynamic doping/dedoping effect of NH_4_^+^ on PANI: PSS.

### 3.3. Detection Principle and Performance Test of Urea Electrode Based on NH_4_^+^ Detection

**Figure 4:**
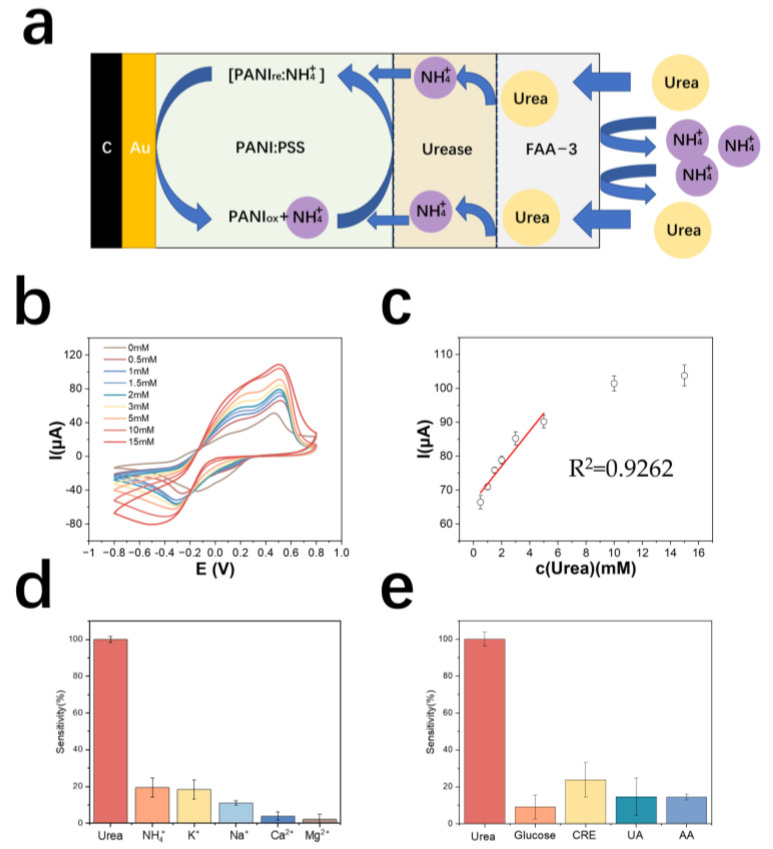
(**a**) Schematic diagram of the detection principle of PANI: PSS-urease-based urea electrode (**b**) cyclic voltammetric curves of urea electrodes in urea solutions of different concentrations (**c**) calibration curves of urea electrodes; (**d**) resistance of urea electrodes to cations (In a 0.01 M PBS solution, sequentially add 5 mM urea, 5 mM NH_4_Cl, 5 mM KCl, 5 mM NaCl, 5 mM CaCl_2_, and 5 mM MgCl_2_); (**e**) resistance of urea electrodes to metabolites (In a 0.01 M PBS solution, sequentially add 5 mM urea, 50 μM glucose, 5 mM CRE, 150 μM UA, and 100 μM AA).

Figure 4a demonstrates the detection principle of the urea electrode. The results of urea detection by cyclic voltammetry are shown in Figure 4b. The detection range of the urea electrode is 0.5~10 mM, the linear range is 0.5~6 mM, and the limit of detection is 500 μM. The equation of the calibration curve is y = 5.20x + 66.66 (y is μA, x is mM), the correlation coefficient is 0.93, and the sensitivity is 106.8 mA M^−1^ cm^−2^ (Figure 4c). Table 2 shows the comparison of electrode properties for urea detection.

The cation resistance of the urea electrode was tested. The results are shown in Figure 4d. The electrode modified with an anion exchange membrane outside the enzyme electrode showed a significantly lower response to NH_4_^+^ in solution. The metabolite resistance of the urea electrode was tested and the results are shown in Figure 4e. The average response of the electrode to interfering creatinine was larger, probably because a small amount of creatinine also passes through the anion membrane and then decomposes in the local acidic environment around the PSS, producing NH_4_^+^, which causes the electrode to react.

### 3.4. Detection Principle and Performance Test of Creatinine Electrode Based on NH_4_^+^ Detection

#### 3.4.1. Performance Testing of Creatinine Electrodes

**Figure 5:**
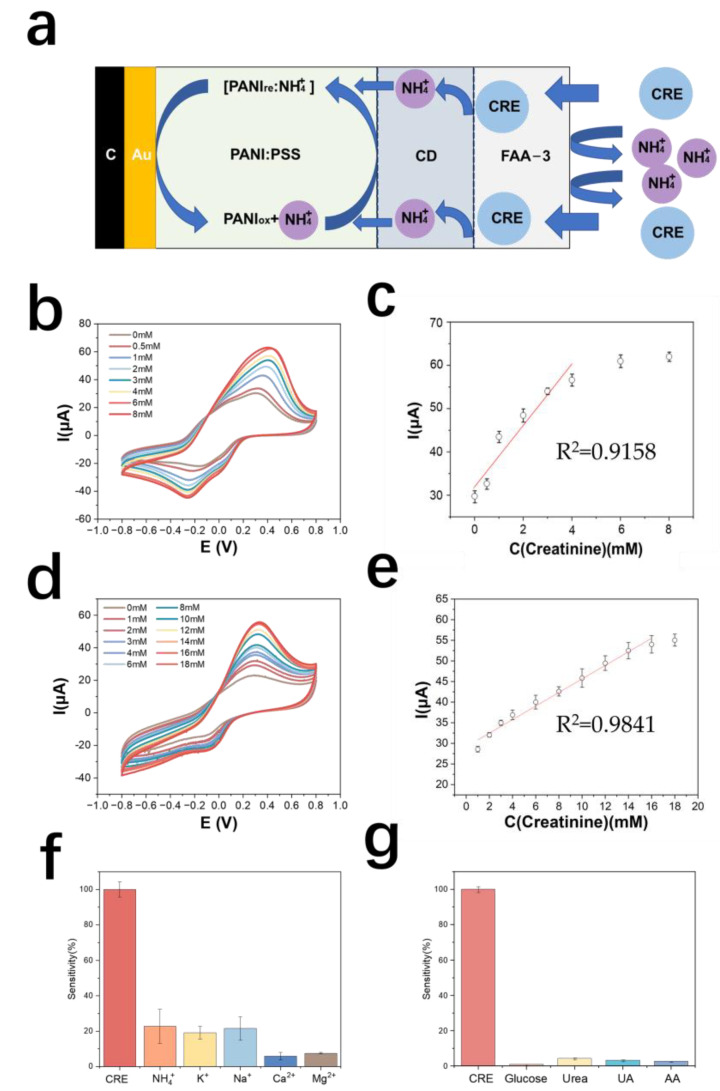
(**a**) Schematic diagram of the detection principle of PANI: PSS-creatinine-deiminase-based creatinine electrode; (**b**) cyclic voltammetric curves of creatinine electrodes in urea solutions of different concentrations; (**c**) calibration curves of creatinine electrodes; (**d**) response curves of chitosan-modified creatinine electrodes for different concentrations of creatinine; (**e**) calibration curves of chitosan-modified creatinine electrodes for different concentrations of creatinine; (**f**) creatinine electrode resistance to cations (In 0.01 M PBS solution, 2 mM creatinine, 5 mM NH_4_Cl, 5 mM KCl, 5 mM NaCl, 5 mM CaCl_2_, and 5 mM MgCl_2_ were sequentially added); (**g**) creatinine electrode resistance to metabolites (In a 0.01 M PBS solution, sequentially add into the solution containing the interfering metabolites, including 50 μM glucose, 10 mM urea, 50 μM UA, and 100 μM AA).

The detection principle of the creatinine electrode is shown in Figure 5a. The test results of the creatinine standard curve are shown in Figure 5b. The detection range of the creatinine electrode prepared by this method was 0.5–6 mM, and the linear range was 0.5–4 mM. The equation of the calibration curve was y = 6.85x + 32.1 (y is μA, x is mM), and the correlation coefficient was 0.91. The sensitivity of this electrode was 139.53 mA M^−1^ cm^−2^ (Figure 5c).

The selectivity results of the creatinine electrode are shown in Figure 5f, in terms of the current response of the electrode to creatinine. The results by metabolite resistance are shown in Figure 5g. The average response of creatinine to other metabolites is less than 5%, indicating that the electrode has good selectivity for creatinine.

#### 3.4.2. Optimization of Creatinine Electrodes

Chitosan has good biocompatibility and is a good material for enzyme immobilization [48]. In this paper, chitosan was used as a material for the diffusion limitation of creatinine. The prepared chitosan solution was 1% chitosan dissolved in 1% acetic acid, and 2 μL of the solution was added dropwise to the enzyme layer of the creatinine electrode with a pipette, dried at room temperature and stored in PBS. Cyclic voltammetry tests for creatinine assay were performed on this electrode and the results are as shown in Figure 5d. The calibration curve of the optimized creatinine electrode assay is shown in Figure 5e. The modification of the chitosan membrane increased the lower detection limit of the electrode from 500 μM to 562.5 μM, the upper detection limit from 6 mM to 18 mM, the sensitivity decreased to 62.34 mA M^−1^ cm^−2^, and the oxidation peak potential decreased from 0.4 V to 0.3 V. It was found that further modification of the anion exchange membrane on the outside of the chitosan layer would lead to electrode failure, presumably because the thickness of the polymer membrane was too thick causing difficulty in diffusion of the substance on the electrode surface and the electrode’s response to creatinine concentration disappears. Therefore, subsequent real sample testing experiments were still performed only using anion-exchange membrane-modified creatinine electrodes. Table 3 shows the comparison of electrode properties for creatinine detection.

### 3.5. Real Sample Testing

Real urine samples were taken for testing the sensor performance, and two urine samples from healthy volunteers, labeled as No. 1 and No. 2, were tested. NH_4_^+^, urea, and creatinine were determined as reference values in urine samples by nano-reagent spectrophotometry and kits, while urine samples were measured using the prepared urine multiparameter detection biosensor. Since the detection range of the prepared urea electrode and creatinine electrode cannot completely cover the concentration range of urea and creatinine in urine, a certain dilution of the real sample is required. The urine samples were accurately diluted 10 times with saline for NH_4_^+^ and creatinine measurements, and 30 times for urea measurements, and the peak oxidation currents obtained from the tests were substituted into the curves tested in artificial urine to obtain the test values. The test results are as shown in Table 4; the relative error of the test results is less than 13%, indicating that the urine multiparameter testing device can be used for diluted sample testing.

## 4. Conclusions

We developed an NH_4_^+^ detection electrode by electropolymerizing PANI: PSS film on a screen-printed carbon electrode, the NH_4_^+^ detection electrode was able to achieve high detection range while maintaining high sensitivity, and the detection mechanism of NH_4_^+^ selective electrode was investigated. On this basis, we immobilized urease and creatinine deaminase on PANI: PSS film to achieve urea and creatinine detection, both of which exhibited good selectivity, consistency, and stability. In addition, we carried out the integration design of the prepared NH_4_^+^, urea, and creatinine electrodes, and fabricated a multiparameter detection device with a paper-based flow channel structure according to the usage scenarios. We tested the real human urine samples, and the test results showed that the urine multiparameter detection device based on the electropolymerized PANI: PSS prepared in this paper could achieve the detection of NH_4_^+^, urea, and creatinine in diluted urine samples. This device provides a platform for the rapid multi-parameter detection of clinical urine and a method for integrated monitoring of other parameters in urine, such as glucose and albumin in the future.

## Figures and Tables

**Table 1:** Comparison of electrode properties for NH_4_^+^ detection.

Electrode Materials	LOD (μM)	Sensitivity (mA M^−1^ cm^−2^)	Linear Range (mM)	Reference
SPE/PANI-Nafion/Cu_2_O/Urease	0.5	250 ± 10	0.001~0.15	[36]
Pt-C/PANI	5	40 ± 20	0.005~1	[42]
PANI-Nafion/Pt	5.35	15.9 ± 0.12	-	[43]
GCE/Nano-PANI: PSS/CPM-Urease	26.9	106 ± 1.8	0.1~11.7	[25]
CD/Nafion^®^-ns PANI/Au/Al_2_O_3_	-	1647 ^a^ 376 ^b^	0.005~1 ^a^ 0.1~0.4 ^b^	[44]
PANI-PSSMA/Au/Al_2_O_3_	-	0.57	1~10	[45]
Nafion^®^(urease)/PANI-Nafion^®^	83	155 ± 6	0.083~1.68	[35]
AuNP/PANI: PSS	290.1	192.6	0.5~20	This work

SPE: Screen-printed electrode; GCE: Glassy carbon electrode; CPM: Capillary pore membrane; ns PANI: nanostructured polyaniline; PSSMA: poly (styrene sulfonate-co-maleic acid, sodium form); AuNP: Gold nanoparticles; ^a^: first linear region in calibration; ^b^: second linear region in calibration.

**Table 2:** Comparison of electrode properties for urea detection.

Electrode Materials	LOD (μM)	Sensitivity (mA M^−1^ cm^2^)	Linear Range (mM)	Reference
SPCE/PANI-Nafion-Cu/Urease	0.5	112 ± 3.36	0.001~0.1	[37]
Nafion^®^(urease)/PANI-Nafion^®^	1 × 10^4^	4.2	-	[35]
GCE/Nano/PANI: PSS/CPM-Urease	51.8	41 ± 5	0.2~0.9	[25]
CNT-SPE/PANI-GND/urease	832.5	22.9	-	[47]
AuNP/PANI: PSS/Urease/FAA	500	106.8	0.5~15	This work

GDN: graphitized nano diamond; FAA: anion-exchange membrane.

**Table 3:** Comparison of electrode properties for creatinine detection.

Electrode Materials	LOD (μM)	Sensitivity (mA M^−1^ cm^−2^)	Linear Range (mM)	Reference
SPCE/PANI-Nafion-Cu/CD	0.5	85 ± 3.4	0.001~0.1	[37]
CANPs/CINPs/SOxNPs/GC	0.01	-	0.00001~0.012	[49]
CD/Nafion^®^-nsPANi/Au/Al_2_O_3_	5	1298.5	0.005~0.4	[44]
AuNP/PANI: PSS/CD/FAA	500	139.53	0.5~4	This work
AuNP/PANI: PSS/CD/Chitosan	562.5	62.34	2~16	This work

SPCE: screen-printed carbon electrode; CD: creatinine deiminase; CANPs: nanoparticles of creatininase; CINPs: nanoparticles of creatinase; SOxNPs: nanoparticles of sarcosine oxidase.

**Table 4:** Urine sample test results.

Sample Number	1			2		
Test Object	NH_4_^+^ (mM)	Urea (mM)	Creatinine (mM)	NH_4_^+^ (mM)	Urea (mM)	Creatinine (mM)
Reference value	26.3	204.7	8.6	31.9	236.7	11.7
Test value	29.7	226.8	9.1	35.8	256.2	12.4
Relative Error	12.9%	10.8%	5.8%	12.2%	8.2%	5.9%

## Data Availability

The data presented in this study are available in article and Supplementary Materials.

## Supplementary Materials

The following supporting information can be downloaded at: https://www.mdpi.com/article/10.3390/bios13020272/s1, Figure S1: Electropolymerization curve of PANI: PSS; Figure S2: Face scan images of elements on the surface of the 12 mg/mL PANI: PSS electrode; Figure S3: Exploration of scanning speed of PANI: PSS electrode; Figure S4: Response of electrode with plating solution containing 2 mg/mL and 60 mg/mL PSS to NH_4_^+^; Figure S5: Effect of deposited AuNPs on electrode generation; Figure S6: Test results of consistency test and stability test; Figure S7: Cyclic voltammetric curves of PANI in different concentrations of ammonium chloride solutions and Cyclic voltammetry curves of PANI and PANI:P SS in 0.01 M PBS; Figure S8: Schematic diagram of the hydrogen ion enrichment effect of PSS; Figure S9: Effect of PH on the detection of NH_4_^+^ by PANI: PSS; Figure S10: Open-circuit potential curve of PANI: PSS with 5 mM ammonium chloride and current curve of PANI: PSS with 5 mM ammonium chloride (−0.2 V constant voltage); Figure S11: Traces of different volumes of red ink in the paper-based flow channel; Table S1: Surface elemental composition of PANI: PSS for different concentrations of PSS; Table S2: Zeta-potential test results; Table S3: Parameter performance comparison of PANI: PSS electrodes with different PSS concentrations; Table S4: Parameter performance comparison of PANI: PSS electrodes with different number of electropolymerization turns.
