# Supplementary material for: Multi-Parameter Detection of Urine Based on Electropolymerized PANI: PSS/AuNPs/SPCE
Source: Biosensors (Basel). 2023 Feb 14;13(2):272. doi: 10.3390/bios13020272 (PMC9954737; doi:10.3390/bios13020272)
Supplement: Supplementary file 1 [file biosensors-13-00272-s001.zip › biosensors-2178265-supplementary.docx]

# Supporting Information

**Electropolymerization of PANI: PSS on AuNPs-modified screen-printed electrode for the detection of ammonium ions, urea and creatinine in urine**

*Dong Wang^1a^, Xiyu Mao^1a^, Yitao Liang^a^, Yu Cai^a^, Tingting Tu^a^, Shanshan Zhang^a^, Tianyu Li^b^, Lu Fang^c^, Yue Zhou^a^, Zhaoyang Wang^a^, Yu Jiang^a^, Xuesong Ye^a^* and Bo Liang^a,d^**

*^a^*Biosensor National Special Laboratory, Key Laboratory of Biomedical Engineering of Ministry of Education, College of Biomedical Engineering and Instrument Science, Zhejiang University, Hangzhou, Zhejiang 310027, PR China

*^b^*Department of General Surgery, Sir Run Run Shaw Hospital, School of Medicine, Zhejiang University,Hangzhou, Zhejiang 310027, PR China

*^c^*College of Automation, Hangzhou Dianzi University, Hangzhou, Zhejiang 310018, PR China

^d^Binjiang Institute of Zhejiang University, Hangzhou 310053, PR China

*^1^*These authors contributed equally to this work.

E-mail: boliang1986@zju.edu.cn (B. Liang), yexuesong@zju.edu.cn (X. S. Ye)


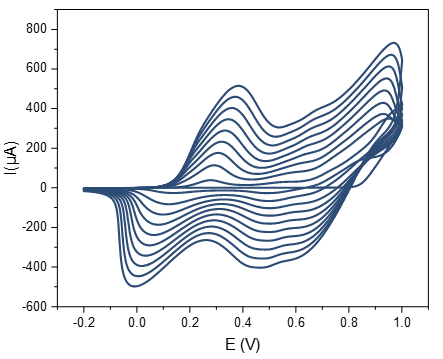


**Figure. S1.** Electropolymerization curve of PANI: PSS.


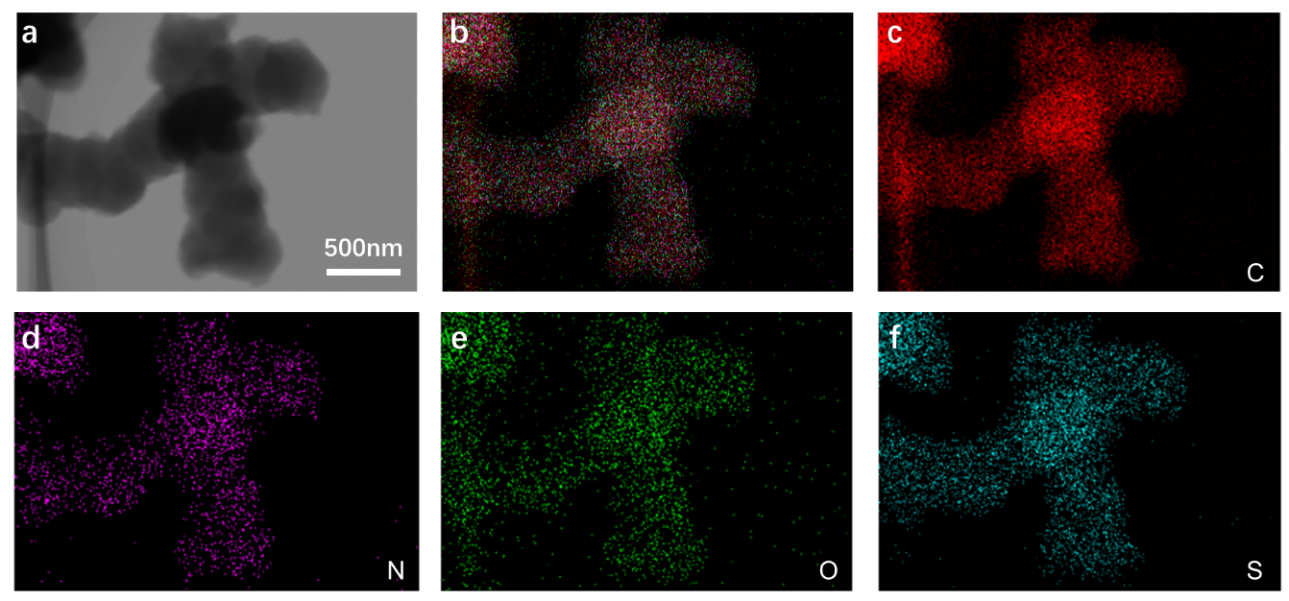


**Figure. S2.** Face scan images of elements on the surface of the 12 mg/mL PANI: PSS electrode, from a to f showing TEM images, images of element combinations, and C (red), N (purple), O (green), and S (cyan), respectively.

The surface scan results show that the four elements are uniformly distributed in PANI: PSS, indicating that the two are doped with each other to form a uniform and stable spherical particle stacking structure during the electropolymerization of PANI.


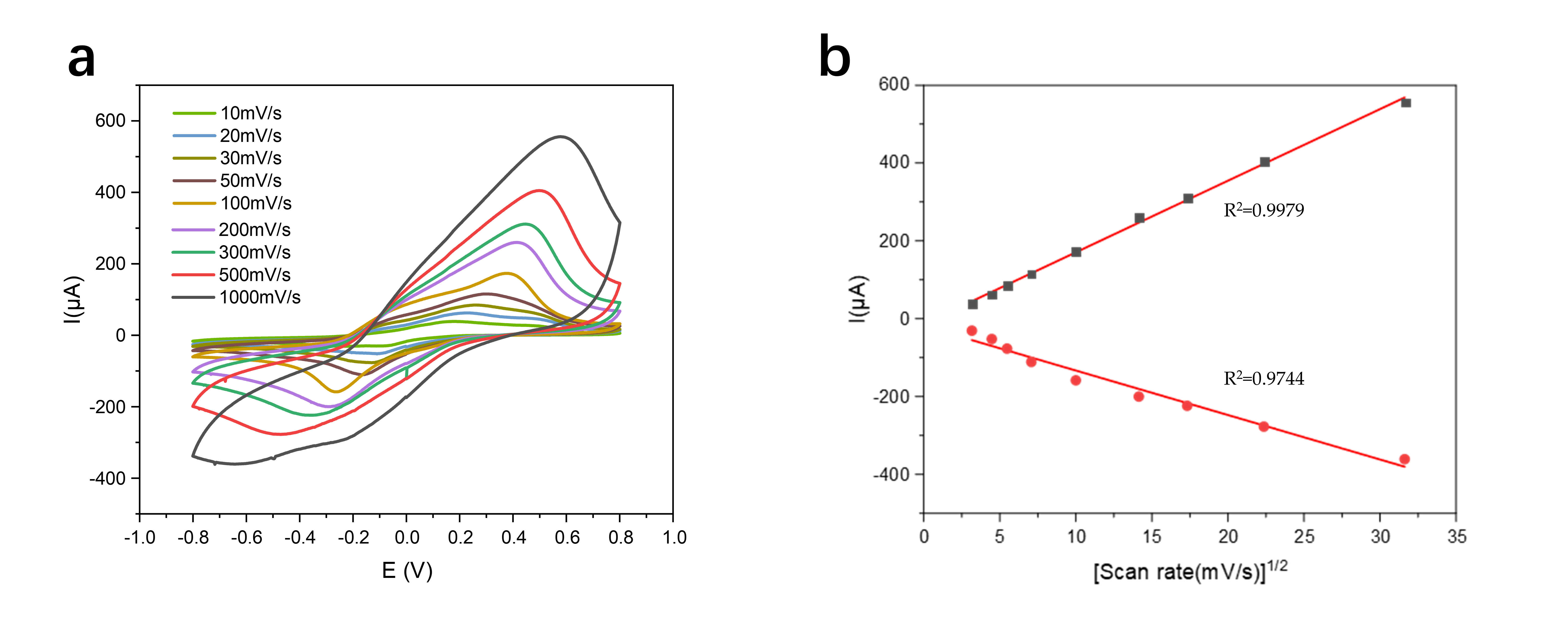


**Figure. S3.** Exploration of scanning speed of PANI: PSS electrode;(a) Cyclic voltammetry curves for different scanning speeds. (b) Redox peak current versus the square root of the scan speed.


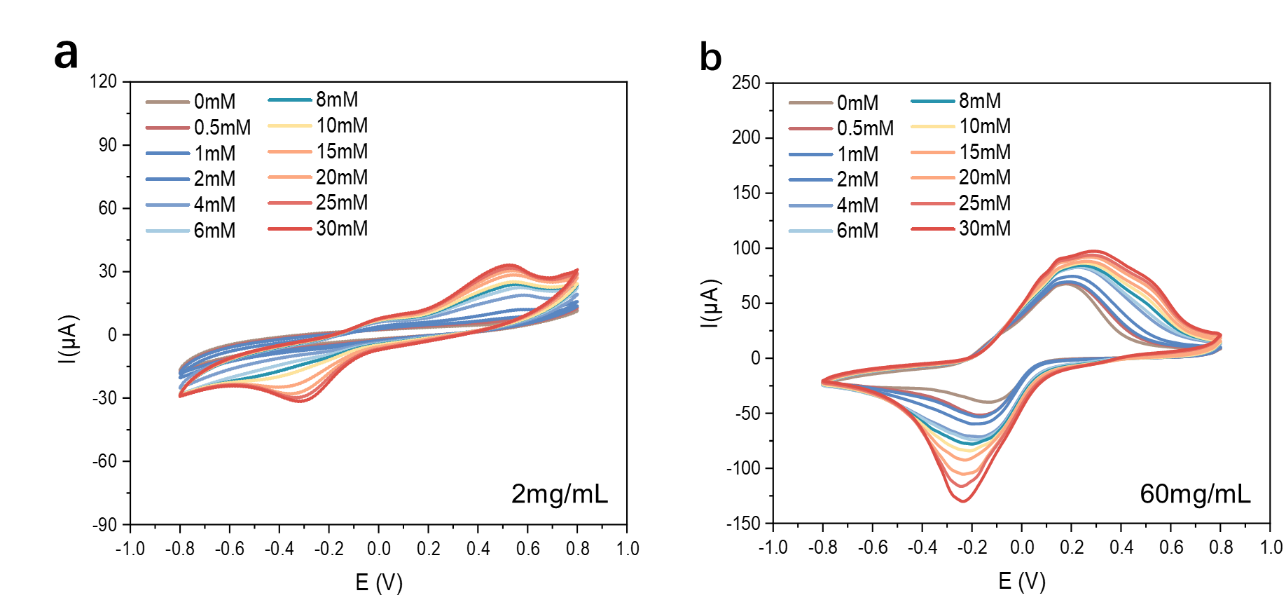


**Figure. S4.** (a) Response of electrode with plating solution containing 2 mg/mL PSS to ammonium ions (The sensitivity is 69.15 mA M^-1^ cm^-2^); (b) Response of electrode with plating solution containing 60 mg/mL PSS to ammonium ions (The sensitivity is 103.94 mA M^-1^ cm^-2^).


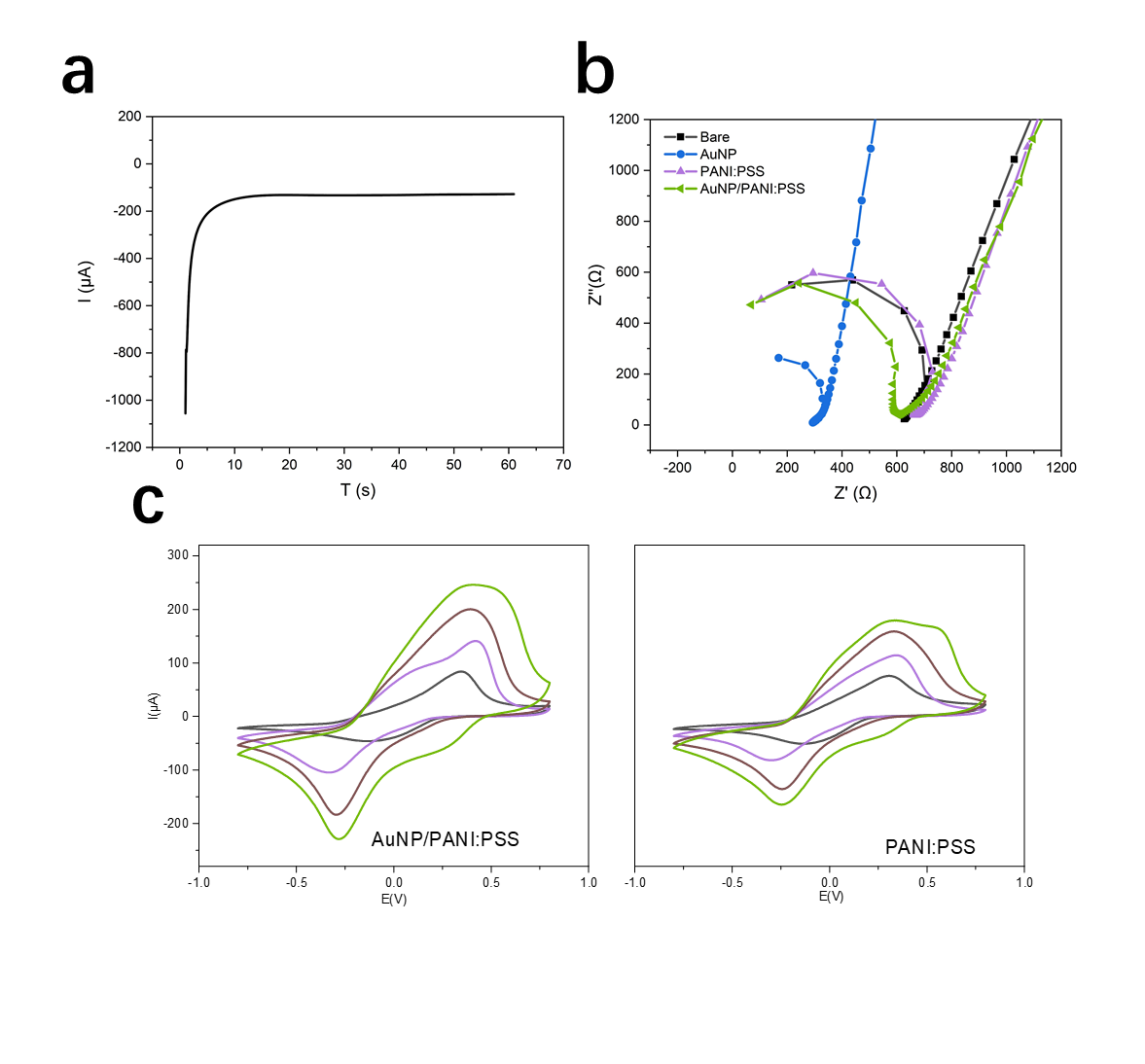


**Figure. S5.** (a)Electrodeposition curve of AuNPs(Reduction of nanogold on electrodes by constant pressure plating in plating solution (0.5 M sulfuric acid solution containing 10 mM chloroauric acid) with specific parameters of 0 V constant potential for 60 s); (b) Impedance test results of AuNP/PANI: PSS(The test solution was an aqueous solution containing 0.1 M KCl and 5 mM K_3_[Fe(CN)_6_]/K4[Fe(CN)_6_] in aqueous solution, the measured frequency range is 10 ~ 106 *Hz* with an amplitude of 10 Mv);(c) Effect of AuNP on the sensitivity of PANI: PSS electrode. (When 1 mM, 5 mM and 10 mM ammonium chloride were added to the solution for testing, the electrode substrate deposited with gold nanoparticles showed a larger cyclic voltammetry current, and the average peak oxidation current increased by about 35%, indicating that gold nanoparticles significantly improved the electron mobility of the electrode and could be used as a material to improve the performance of the electrode. All the subsequent experiments used AuNP/PANI: PSS electrodes)

As shown in **Fig. S5**c, the electrode deposited with gold nanoparticles on the electrode substrate exhibited a larger cyclic voltammetric current with an increase in the average oxidation peak current of about 35%, indicating that gold nanoparticles have a significant increase in electron mobility for the electrode and can be used as a material to improve the electrode performance.


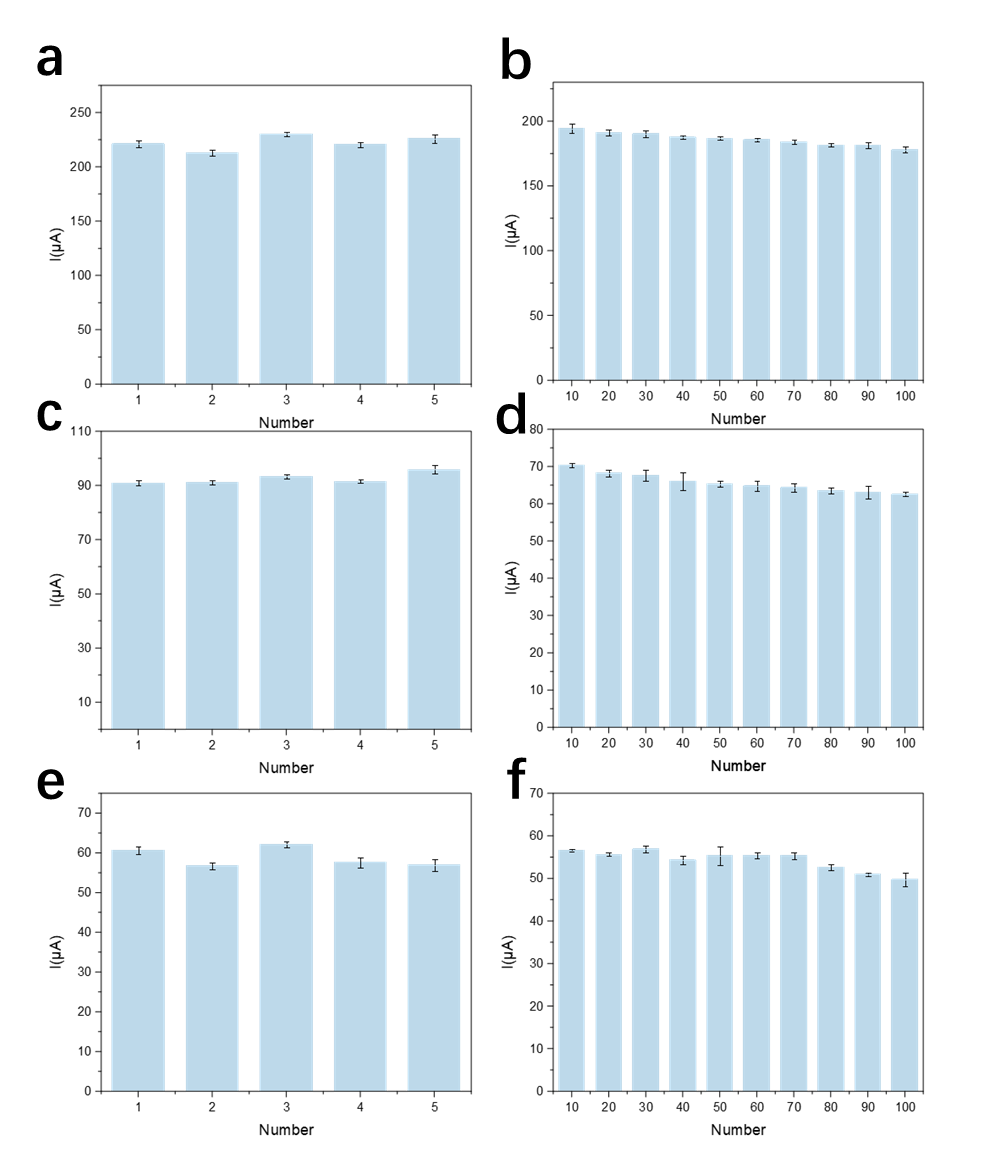


**Figure. S6.** (a) consistency of AuNP/PANI: PSS electrode; (b) ammonium ion selective electrode stability; (c) consistency of urea electrodes; (d) stability of urea electrodes; (e) consistency of creatinine electrodes; (f) stability of creatinine electrodes

**Consistency testing**: Five ammonium ion/urea/electrodes were prepared by the same electrode preparation method, and cyclic voltammetry tests were performed in 5 mM ammonium chloride, 5 mM urea, and 2 mM creatinine solutions, respectively, and the peak oxidation currents of the electrodes were taken for graphing.

**Stability testing:** The three kinds of electrodes were subjected to 100 cycles of voltammetry tests in 0.01 M PBS solutions containing 5 mM ammonium chloride, 5 mM urea, and 2 mM creatinine separately, and the average of the peak oxidation currents for each 10 scans was taken for graphing.


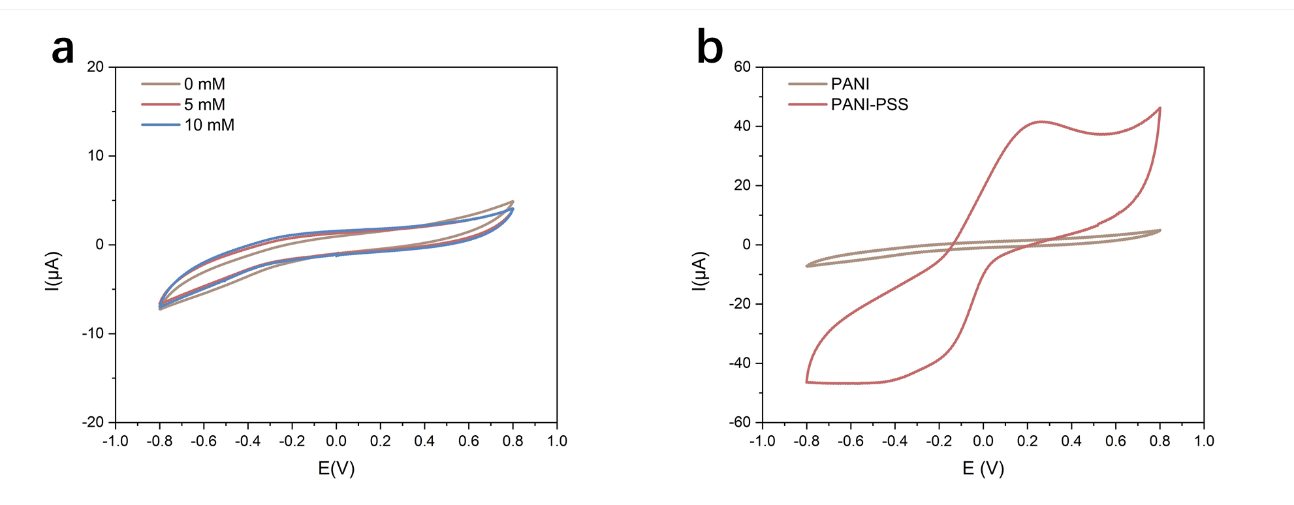


**Figure. S7.** (a) Cyclic voltammetric curves of PANI in different concentrations of ammonium chloride solutions; (b) Cyclic voltammetry curves of PANI and PANI:P SS in 0.01M PBS.


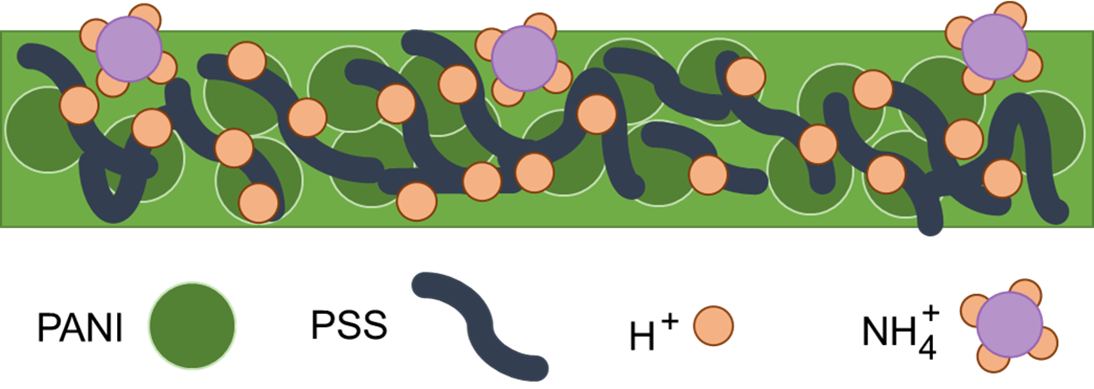


**Figure. S8.** Schematic diagram of the hydrogen ion enrichment effect of PSS.


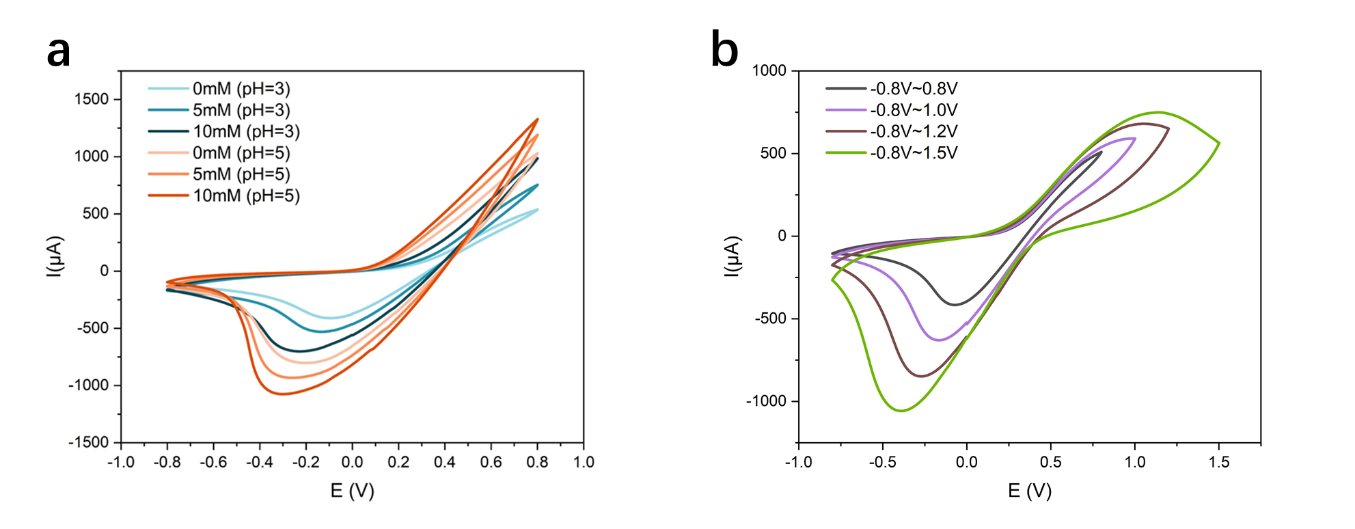


**Figure. S9.** (a) Cyclic voltammetric response of PANI to ammonium ions in McIlvaine buffer solution. (b) Cyclic voltammetric response of PANI to 5 mM ammonium chloride in pH = 3 solution (upper limit of extended oxidation potential);


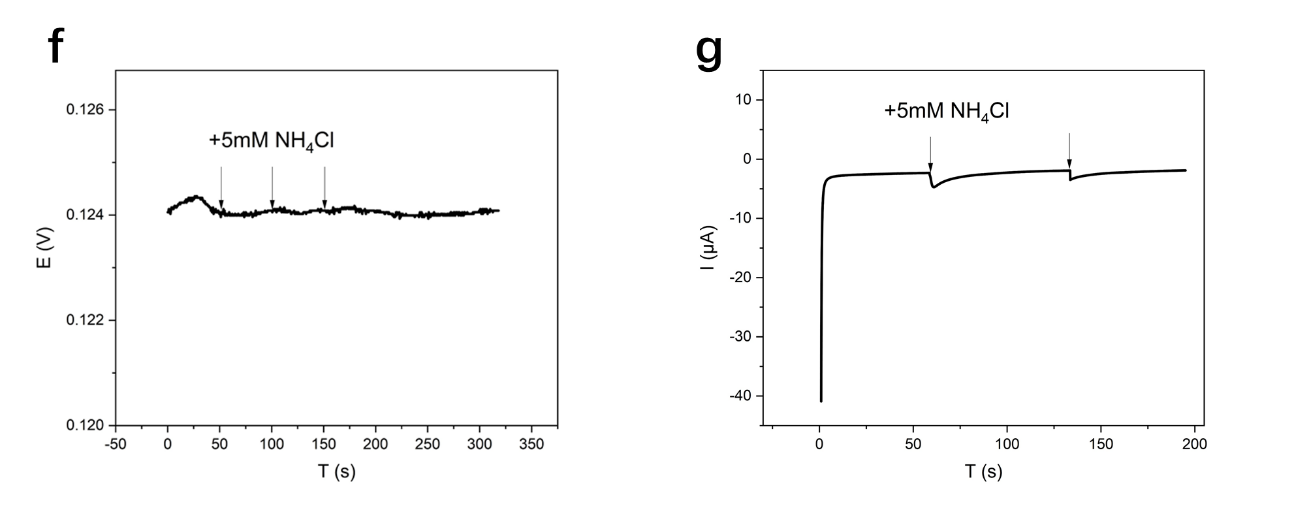


**Figure. S10**. (a) open-circuit potential curves of PANI: PSS; (b) current curves of PANI: PSS (-0.2 V constant voltage).

**
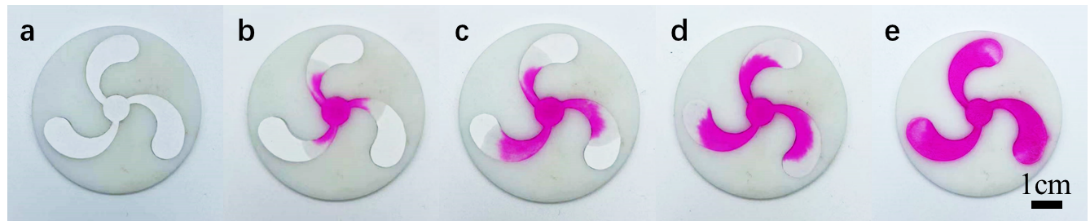
**

**Figure. S11.** Traces of different volumes of red ink in the paper-based flow channel. (a) 0 μL, (b) 20 μL, (c) 40 μL, (d) 60 μL, and (e) 100 μL.

Point-of-care testing (POCT) is a method that can be performed without the need for specialized laboratory personnel and specialized instruments[2]. No or simple pre-treatment of the sample is required before testing, and its use includes disease management, health status tracking, or environmental monitoring, with results available within hours or seconds[3].Accordingly, we designed a urine multiparameter assay device with three recesses for ammonium ion, urea and creatinine electrodes, respectively. In order to ensure that the sample liquid can adequately cover the detection area of the electrodes, we made a structural verification of the paper-based flow channel of the assay device, whose liquid transport characterization is shown in **Fig. S11**, using diluted red ink to observe the flow of the sample in the paper-based flow channel more clearly. After adding 20 μL of red ink drops to the sample inlet every 10 s, it can be seen that the filter paper rapidly absorbed the red ink and transported the liquid along the three fan blade directions, and after adding 60 μL drops, the water trace reached the end of the fan blade which is the electrode detection region. It is noteworthy that the red ink leaves two traces on the filter paper, the water trace as well as the red ink trace. The water diffuses further than the red ink, which demonstrates the chromatographic properties of the filter paper, where different molecular weights of substances in the solution diffuse at different rates, with smaller molecules being able to diffuse further. After 100 μL drops were added, the fan leaves were completely covered by red ink and a small amount of liquid touching the detection electrode could already be observed in the detection area. In order for the sample to completely cover the detection electrodes to achieve a stable detection environment, it was tested that about 500 μL of liquid was needed to make the sample completely cover the three detection electrodes, which is suitable for micro urine analysis scenarios.

**Table S1.** Surface elemental composition of PANI: PSS for different concentrations of PSS

| Element | Percentage of atoms（%） | | | |
| --- | --- | --- | --- | --- |
|  | 0 mg/mL（pure PANI） | 2 mg/mL | 12 mg/mL | 60 mg/mL |
| C | 89.48 | 87.45 | 80.48 | 78.68 |
| N | 7.46 | 3.11 | 7.24 | 10.39 |
| O | 2.99 | 8.89 | 10.30 | 8.09 |
| S | 0.07 | 0.55 | 1.98 | 2.84 |

**Table S2.** Zeta-potential test results

| Material | zeta-potential（mV） |
| --- | --- |
| PANI | + 23 |
| PSS | - 40 |
| PANI: PSS | - 30 |

**Table S3.** Parameter performance comparison of PANI: PSS electrodes with different PSS concentrations

| PSS concentration of electroplating solution (mg/mL) | Sensitivity (mA M^-1^ cm^-2^) | Testing range (mM) | Detection limit (mM) |
| --- | --- | --- | --- |
| 2 | 69.15 | 2 ~ 30 | 2 |
| 12 | 192.6 | 0.5 ~ 40 | 290.1 |
| 60 | 103.94 | 1 ~ 30 | 1 |

**Table S4.** Parameter performance comparison of PANI: PSS electrodes with different number of electropolymerization turns

| Number of cycles of voltammetric plating | Sensitivity mA M^-1^ cm^-2^ | Testing range (mM) | Detection limit  (mM) |
| --- | --- | --- | --- |
| 5 | 55.04 | 2 ~ 30 | 2 |
| 10 | 192.6 | 0.5 ~ 40 | 290.1 |
| 20 | - | - | - |

**References:**

1. Zhou, W.; Gao, X.; Liu, D.; Chen, X. Gold Nanoparticles for in Vitro Diagnostics. *Chemical Reviews* **2015**, *115*, 10575–10636, doi:10.1021/acs.chemrev.5b00100.

2. Gubala, V.; Harris, L.F.; Ricco, A.J.; Tan, M.X.; Williams, D.E. Point of Care Diagnostics: Status and Future. *Analytical Chemistry* **2012**, *84*, 487–515, doi:10.1021/ac2030199.

3. Meagher, R.J.; Hatch, A. V.; Renzi, R.F.; Singh, A.K. An Integrated Microfluidic Platform for Sensitive and Rapid Detection of Biological Toxins. *Lab on a Chip* **2008**, *8*, 2046–2053, doi:10.1039/b815152k.
